# Households, bubbles and hugging grandparents: Caring and lockdown rules during COVID-19

**DOI:** 10.1007/s10691-020-09445-z

**Published:** 2020-11-23

**Authors:** Jackie Gulland

**Affiliations:** School of Social and Political Science, University of Edinburgh, Chrystal Macmillan Building, 15a George Square, Edinburgh, EH8 9LD UK

**Keywords:** Care, COVID-19, Ethics of care, Gender, Households, Interdependency

## Abstract

Efforts to combat the COVID-19 crisis brought mountains of legislation and guidance to coerce or encourage people to stay at home and reduce the spread of the virus. During peak lockdown in the United Kingdom (UK) regulations defined when people could or could not leave their homes. Meanwhile guidance on social distancing advised people to stay within ‘households’. This paper explores the legislation under lockdowns in the UK from March to October 2020 and the implications for women’s gendered caring roles. The regulations and guidance assumed that households were separate units and ignored the interdependencies which exist between households and between individuals and wider society. The continuing focus in the lockdown regulations has been on households as autonomous, safe, adequate and secure. This overlooks the interdependency of human life, gendered aspects of caring and the inequalities of housing and living conditions, highlighted by feminist scholarship.

## Introduction

In March 2020 the UK entered a period of lockdown, to tackle the COVID-19 crisis.^1^ Legislation during the peak lockdown period (March–May 2020) required most people to stay at home and ordered most workplaces and public services to close down. Formidable powers were given to governments and local authorities to control public order and restrict freedom of movement, creating major threats to civil liberties across the spectrum (for details see Greene 2020, Liberty 2020a). Lockdown brought immediate pressures on the everyday workings of society. With the closure of schools, the term ‘home schooling’ was used by policymakers and the press to describe how parents should organise their days. While parents in middle class jobs struggled to look after their children while working from home, this was less feasible for people in frontline working class jobs, and so they risked losing their income, risking their own health and struggling to provide care and education for their children (Warren and Lyonette 2020). Parents of disabled children had additional challenges brought about by the loss of existing childcare support systems, home schooling and concerns about their children’s health. Early research on the effects of lockdown on parents showed that women carried the bulk of this childcare work, at the expense of their ability to continue in paid work and with substantial impacts on their mental health (Collins et al. 2020; Craig and Churchill 2020; Engender 2020; Rose-Redwood et al. 2020). Meanwhile those providing care for adults found that many formal and informal supports disappeared, leaving them isolated and exhausted.

In this paper I will show that a feminist analysis of the lockdown rules exposes neo-liberal assumptions about the family household as autonomous and sufficient for the provision of reproductive labour. Feminists have long noted that reproductive labour has been, and continues to be, heavily gendered, with women continuing to carry out the bulk of childcare, housework and adult care (Huws 2019). Feminist and disability scholars question neo-liberal ideas about autonomy and emphasise the interdependency of human life (Morris 1991; Witcher 2015). A feminist ‘ethics of care’ recognises this interdependency and that care is fundamentally relational (Barnes 2012). In this paper I will show how the failure by policymakers to take account of this interdependency has made lockdown more difficult for carers and those in receipt of care. This burden has fallen on women and on low paid, working class and black and minority ethnic women in particular.

Before considering the lockdown rules, it is important to note the unequal impact of COVID-19. There is growing evidence that the greatest health impacts of COVID-19 have been on those in the poorest areas of the country, particularly on black and minority ethnic communities and that there are clear relationships between existing structural health inequalities and the effects of the virus. Evidence from disability organisations, older people’s groups and carers’ organisations shows that life has been particularly difficult under lockdown. Emerging findings show that women have been particularly badly affected by the social consequences of lockdown across a range of issues.

## UK regulations on lockdown

The law covering the lockdown in the UK was defined in regulations under the Coronavirus Act 2020 and the Coronavirus (Scotland) Act 2020, with separate regulations in each of the four jurisdictions of England, Scotland, Wales and Northern Ireland. Lockdown has, at the time of writing, been in three phases: ‘peak lockdown’ from 26 March to May 2020; relaxed lockdown during the summer of 2020; and a period of increased restrictions and local lockdowns from the autumn of 2020. Much of the direct effect of the legislation concerned closure of business and services, which had a knock on effect on earnings and on support services. The effect of the legislation on individual behaviour was more indirect, with government guidance and persuasion used as key tools rather than direct enforcement. Police powers to fine individuals in breach of lockdown rules tended to be enforced for those thought to be out and about in public in breach of the regulations, rather than for those breaching rules within the private sphere. An investigation by Liberty showed that fines for breaches of lockdown were used disproportionately against people from black and minority ethnic groups (Liberty 2020b).

However, even where regulations were not enforced, the use of language in law has important symbolic effects (Levitsky 2014) and it is worth investigating how some of the terms used in the legislation both reflect and affect gendered caring roles. Concepts which have been key to domestic and caring arrangements during lockdown include the terms ‘households’ and ‘linked’ or ‘extended’ households, popularly known as ‘bubbles’. Before moving on to look at the role of households under lockdown, it is important to consider the details of the lockdown legislation and the definitions of these terms.

In each of the four jurisdictions, regulations during the peak lockdown period stated that people must not leave their homes except in certain very specific circumstances. Breaching these regulations constituted an offence, although there was a range of important exceptions to the requirement to stay at home, the main ones being:

- To shop for basic necessities
- To take exercise
- To seek medical assistance
- To provide care or assistance to a vulnerable person
- To travel to work

The regulations assumed that most ‘work’ was carried out in the workplace and that workers had no other responsibilities or commitments. The concept of the household was important, not so much for the restrictions on leaving home but in the regulations regarding ‘gatherings’ in public places, where people were not permitted to gather in a public place unless they were members of the same household, or under certain other conditions, including “providing care or assistance to a vulnerable person”.^2^ ‘Household’ was not defined in the regulations in the spring but guidance suggested that the interpretation was relatively broad and so did not fall into the trap of defining members of households by their legal status, for example in relation to marriage, cohabitation, civil partnership or formal tenancy agreements. Amendments to regulations in the autumn did provide definitions of ‘household’, generally defining a household as people living at the same address.^3^ Government guidance used the term ‘household’ frequently in relation to the advice about social distancing, for example in relation to the ‘two metre’ rule when interacting with people not in the same household or in relation to the advice on quarantine if someone in the household had symptoms of the virus (UK Government 2020). Household was therefore an important category in policy discourse about the lockdown. The rules on households were relaxed in early summer (at different times in each jurisdiction) to enable different households to create what were known as ‘linked households’ in England and Northern Ireland or ‘extended households’ in Wales and Scotland. The popular term for these was ‘bubbles’, with the verb ‘bubbling’ appearing in some guidance (for example NI Direct 2020).

## Households

Further examination of these concepts from a feminist perspective helps to reveal the underlying assumptions in the lockdown regulations. The idea of ‘household’ implies that homes are safe, secure and that there is sufficient space for everyone to isolate together. Early findings from research on the lockdown shows that access to space is unequal, with those from wealthier, white households more able to self-isolate and work at home while those with lower incomes and from black, Asian and minority ethnic backgrounds less able to do so (Atchison et al. 2020). This appeared to be one of the key factors in the unequal death rates of those from black, Asian and minority ethnic backgrounds (Public Health England 2020). The focus on households as the key category for social distancing measures meant that those whose household arrangements were not safe were trapped. Early findings from research on the effects of this suggests that women and children experiencing domestic abuse were particularly vulnerable (Green 2020). Some LBQTI people were forced to share households where their identities were at risk, for example when young people were forced to move back in with their parents (Kneale and Becares 2020).

The idea of the household also assumed that small groups of people or single people could exist in isolation from other households. Although the definition of household was broad and was not restricted to heterosexual nuclear families, the concept built on the idea of the family as a safe and sufficient space, a concept which feminists have long challenged (Sinha 2013).

The exceptions allowing people to leave their homes during peak lockdown were very narrow, with the priorities being work, healthcare, essential shopping, exercise and supporting others who were defined as ‘vulnerable’. ‘Vulnerable’ was defined in the regulations in terms of susceptibility to particular harm from the virus, including those aged over 70 and those with a range of ‘underlying health conditions’.^4^ Although this exception provided some protection for those most susceptible to harm from the virus, many disabled and older people’s organisations objected to the use of the term ‘vulnerable’ as demeaning and failing to recognise the value of disabled and older people to society. Disability activists have challenged the idea of disabled people as exceptionally vulnerable (Scully 2013), while others have call on an ethics of care which embraces the vulnerability of all human life (Barnes 2012).

The narrow definition of ‘vulnerable’ in terms of susceptibility to the virus also excluded many people who were not medically vulnerable but who might need support from others, for example many people with cognitive impairments or mental health issues. There was no provision for the myriad informal networks of support which many disabled people, parents and carers rely on in normal times.

## Bubbles

The rules on households were relaxed in early summer to enable different households to create what were known as ‘linked households’ in England and Northern Ireland or ‘extended households’ in Wales and Scotland. This meant that people who lived on their own or only with children under 18 could create a ‘bubble’ with another household, enabling them to spend time in each other’s houses without having to follow social distancing rules. They could also do things together in public without falling foul of the rules on public gatherings. For example the guidance in Scotland stated:

Once two households have agreed to form an extended household they may meet outdoors or indoors, visit and stay at each other’s homes, and do everything that people in other households can do, such as watch TV, share a meal and look after each other’s children. (Scottish Government 2020)

The social bubbles were introduced in an attempt to combat the loneliness that many people had experienced during peak lockdown. However the guidance was complex. For an attempt to explain it, see Roberts (2020). The idea of bubbles was welcomed at the time by the media as providing an opportunity at last for grandparents to hug their grandchildren. Some commented on the irony that this would only work if either the children lived with a lone parent or if the grandparents were themselves single. Children in households consisting of two adults would only be able ‘hug their grandparent’ if the grandparent lived alone. The idea that the grandparents themselves might have anything to contribute through the relaxation of household rules was seldom mentioned. In normal times grandparents often provide the daily childcare which enables parents to engage in the labour market (Kanji 2018), while a smaller but important number act as kinship carers for their grandchildren, having full time responsibility for them (Hunt 2018).

An irony for many was that people who lived together could not form social bubbles with other households. People living in households where there were difficult relationships or particular stresses could not seek emotional support from other friends or relatives (except remotely). Similarly, people experiencing mental distress or domestic abuse but who happened to live in a household with other adults would not necessarily find any relief from their isolation. As the regulations changed over the summer the definitions of bubbles changed in each of the four jurisdictions, so that in Northern Ireland and Wales, the requirement was removed for one household to be a single person or lone parent.^5^ However, the principle remained that each household could only be in one bubble and that all adult members of each household would need to agree about this. Again, this assumed that a household is a happy and safe place. I now turn to how the household and bubbles rules affected care relationships, first of all, childcare and then adult family care.

## Childcare

The policy rhetoric on school closures during peak lockdown was almost entirely about the effect on children’s education, something that could be addressed by ‘homeschooling’ but the role of schools as providers of childcare was somewhat muted. There was even less discussion in the media about the informal childcare provision such as childminders, or family networks of support on which many parents rely. Much of this informal support is provided by grandparents, other family members and mutual arrangements between friends and colleagues (Raw and McKie 2019; Hill et al. 2020). That support was not available so long as households were required to remain apart during lockdown.

The regulations assumed that care could be provided within the household unless a parent was attending work as a ‘key worker’ and was making use of formal childcare. The regulations were amended after the public outcry when the senior government advisor, Dominic Cummings, allegedly breached the regulations in order to seek care for his child (Dearden 2020). The amended regulations defined ‘childcare’ as equivalent to that under the Childcare Act 2006, essentially ruling out any informal childcare arrangements between relatives.^6^

When the lockdown eased in early summer, it became possible to use ‘bubbles’ for childcare support, although the guidance warned that this should not be used with more than one other household:

If you are a lone parent you can form a support bubble with another household to provide informal (unpaid) childcare for them or for them to provide informal childcare for you. You should not form a support bubble with more than one household. (Department of Health and Social Care 2020)

The idea that informal childcare could be provided only within bubbles was continued when greater restrictions were introduced in the autumn, for example, in the regulations regarding ‘Tier 3′ lockdown in England, which introduced the idea of ‘childcare bubbles’ where parents could create bubbles with one other household for the purposes of childcare.^7^ They would not, however, be able to form other bubbles for other purposes, for example to support adult care.

## Adult family carers

While the mainstream media noted the problem with childcare, there was little discussion of the role of care in adult relationships. Many services for older people, disabled people and carers provided by local authorities and third sector agencies disappeared overnight, while some agencies struggled under the increase in staff absences while staff self-isolated (Carers UK 2020a, b; Inclusion Scotland 2020). The Coronavirus Act 2020 amended social care legislation across the UK so that local authorities’ duty to assess needs, provide social care services and support carers was relaxed to enable local authorities to focus on areas of most urgent need^8^ as well as amending provision under mental health legislation (Vicary et al. 2020). At the same time, a myriad of voluntary community and charitable organisations increased their services to provide food parcels, medication delivery services and social support, within strict limits. The regulation permitting people to leave home for work included permission to travel ‘to provide voluntary or charitable services’,^9^ a recognition of the need for structured charitable or community support but not for the kind of everyday informal support provided by networks of family members and friends.

During peak lockdown many family carers supported family members while maintaining strict isolation. People needing support are sometimes those most likely to be considered to be medically ‘vulnerable’ and many were in the group of people who should have been ‘shielded’, according to the government guidance. This meant that some disabled people and carers could not leave their homes but also that they had to pay particular attention to safety and protection if anyone entered their homes. Some chose to isolate completely and to stop all visits from people who might support them. Others had no choice but to risk exposure to the virus, with inadequate protection equipment. Some families chose to move in together to form a household in order to provide the support they needed, for example young disabled people moved in with parents, thus giving up their independence which they had fought hard to gain. Early research by disabled people’s and carers’ organisations showed that disabled people and carers experienced extreme deprivation and distress during peak lockdown (Carers UK 2020a, Inclusion Scotland 2020; Miller 2020). Social isolation has risked people’s physical health, through lack of access to food and to exercise and has also compounded problems of mental wellbeing for those who were already isolated. Meanwhile people working in the frontline of paid care work, disproportionally women and people from black, Asian and minority ethnic backgrounds, continued to risk their own health while continuing to provide care services, often without adequate protective equipment. The guidance on households exacerbated the isolation of people who were already isolated because they were disabled or by their caring roles.

Adult social care relies on networks of individuals. Research by Bowes et al. (2020) on time and caring illustrates the diversity of caring tasks, including socialising, dealing with bureaucracies and supporting people to manage everyday life. Care relationships are interdependent and they happen across households. Although family carers are the most common relationship, care relationships also happen between friends and neighbours. A report by Carers UK, six months into lockdown provides evidence of how some of these networks have collapsed (Carers UK 2020b). The report provides vivid examples of how simple things such as the loss of local day services, or the opportunity to meet up with friends or other family members can have devastating effects on carers’ mental wellbeing.

## Care and feminist theory

Feminists have long grappled with the concept of ‘care’. Harding et al. (2017) argue that there have been three themes in this scholarship: first of all that ‘care’ should be recognised as an aspect of unpaid domestic labour; secondly, following Sevenhuijsen’s ‘ethic of care’, that ideas of care must include the moral dimension of care relationships; and more recently, a re-emphasis on the value of being ‘cared for’ (Harding et al. 2017, 1). This refocus on the interdependency of care relationships builds on disability rights theorists, who often reject the term ‘care’, as demeaning. Disability rights theorists have been strong advocates for understanding the interdependency of human beings (Clough 2014; Witcher 2015; Harding 2017), while feminists and carers’ rights theorists have shown that care relies on networks of care beyond the individual and beyond traditional ideas of ‘households’ (Sevenhuijsen 2003; Barnes 2012; Fraser 2016; Bowlby and McKie 2019). Levitsky reminds us that most caring continues to be considered a family responsibility and that the gendered assumptions about who provides this care and in what circumstances are often hidden from view (Levitsky 2014). Most care work, paid and unpaid, is done by women. However, looking through a feminist lens, which helps us to see care as interdependent and relational, helps to value the care done by men too.

In this paper I have looked at the way in which the COVID-19 lockdown regulations in the UK have been constrained by assumptions that care happens either in the government, private and charitable care sectors or that it can be contained within a household. As the regulations changed over the first six months of the crisis, there was some relaxation of rules about households to enable informal childcare provision and to address the problem of loneliness for people living alone. The continuing focus in the lockdown regulations has been on households as autonomous, safe, adequate and secure, disguising the interdependency of human life, gendered aspects of caring and the inequalities of housing and living conditions.

## A final reflection

There is a personal angle to this. I started writing this piece in response to my own position as an informal carer for someone with dementia. Peak lockdown for me was characterised by daily worry about whether my relative would get the support she needed, whether she was safe, whether the low paid care staff who visited her daily were safe, whether I should visit or not, whether other family members should visit or not, whether we could go out for a walk together, whether any of us constituted an extended household. I worried that the loneliness and social isolation would make things worse. As the crisis continued, some of those worries abated and I became more confident that I could continue to support my relative. Since she lives on her own we are, perhaps, an extended household. We have been fortunate in that formal social care provision was not reduced and was in fact increased when new support needs emerged during peak lockdown. However, other informal supports disappeared and my relative’s social networks vanished overnight. Technology has not provided a solution. Hugging grandchildren is not an option. My own position as a white, middle class woman who can work from home makes this task easier compared with many but I am not certain that having a professional interest in gender, law and social inequality has helped at all.

## Footnotes

1 This article concerns the UK, but many of the gendered patterns of COVID-19 and associated restrictions can be seen across other countries, see for example McLaren et al. (2020), Brennan et al. (2020).

2 The Health Protection (Coronavirus) (Restrictions) (Scotland) Regulations (2020), 6.

3 For example in The Health Protection (Coronavirus) (Restrictions and Requirements) (Scotland) Regulations (2020), introduced in October 2020.

4 The Health Protection (Coronavirus) (Restrictions) (Scotland) Regulations (2020), 10.

5 The Health Protection (Coronavirus Restrictions) (No. 2) (Wales) Regulations 2020; The Health Protection (Coronavirus, Restrictions) (No. 2) (Amendment No. 9) Regulations (Northern Ireland) 2020.

6 The Health Protection (Coronavirus, Restrictions) (England) (Amendment) (No. 3) Regulations 2020.

7 The Health Protection (Coronavirus, Local COVID-19 Alert Level) (Very High) (England) Regulations 2020, Schedule 1, para 8.

8 Coronavirus Act 2020, s15, 16.

9 The Health Protection (Coronavirus) (Restrictions) (Scotland) Regulations (2020), 8(5)(f).
